# One-Pot LAMP-Coupled CRISPR/Cas12b Assay Enables Sensitive Detection of *Helicobacter pylori*

**DOI:** 10.3390/biology15100797

**Published:** 2026-05-16

**Authors:** Ziyan Tang, Wentao Bai, Shuting Yan, Gaoming Luo, Yanheng Zheng, Zhuojun Bai, Zhu Chen

**Affiliations:** 1 MOE Key Lab of Rare Pediatric Diseases, Hengyang Medical College, University of South China, Hengyang 421001, China; 13807432359@163.com (Z.T.);; 2 School of Electrical Engineering, University of South China, Hengyang 421001, China; 3 Health Management Center, Zhuzhou Hospital Affiliated to Xiangya School of Medicine, Central South University, Zhuzhou 412007, China; 4 Institute for Future Sciences, University of South China, Changsha 410008, China

**Keywords:** *H. pylori*, LAMP, CRISPR/Cas12b, *CagA* gene, point-of-care testing

## Abstract

*Helicobacter pylori* (*H. pylori*) infection is closely associated with the development of chronic gastritis, peptic ulcers, and gastric cancer, highlighting the importance of rapid and accurate detection for disease prevention and clinical management. In this study, a one-pot LAMP-CRISPR/Cas12b assay targeting the *CagA* gene was developed for *H. pylori* detection. First, the LAMP system was optimized by systematically screening key reaction components. Subsequently, a one-step LAMP-CRISPR/Cas12b detection platform was established through optimization of the ratio between the LAMP premix and CRISPR buffer, reaction temperature, Cas12b concentration, and ssDNA reporter concentration. Under optimal conditions, the assay achieved a detection limit of 3.14 × 10^1^ copies/µL, representing a tenfold improvement in sensitivity compared with conventional LAMP and PCR assays (3.14 × 10^2^ copies/µL). In addition, the entire detection process could be completed within 1 h. Validation using 17 culture-positive and 17 culture-negative samples demonstrated complete concordance with culture-based results, with no false-positive or false-negative detections observed. These findings indicate that the proposed platform possesses high sensitivity, excellent specificity, rapid turnaround, and operational simplicity, demonstrating strong potential for point-of-care testing and applications in resource-limited settings.

## Simple Summary

Early and efficient detection of *Helicobacter pylori* is critical for gastric cancer prevention, particularly in resource-limited settings. Current diagnostic methods, however, remain inadequate for rapid, on-site testing. Here, we developed a one-pot LAMP-CRISPR/Cas12b system targeting the conserved *CagA* gene, combining rapid isothermal amplification with Cas12b-mediated signal amplification. The assay can be completed within one hour, achieves a tenfold higher sensitivity compared to quantitative polymerase chain reaction or loop-mediated isothermal amplification alone, and shows no cross-reactivity with other pathogens. This method provides a powerful tool for point-of-care testing and clinical diagnosis of *Helicobacter pylori*.

## 1. Introduction

*H. pylori* is a Gram-negative, microaerophilic, spiral-shaped bacterium recognized as one of the most common human pathogens responsible for chronic infections worldwide. It has a widespread distribution and poses considerable health risks. As of 2015, approximately 4 billion people globally were estimated to be infected, with distinct geographic disparities in prevalence: at the turn of the 21st century, infection rates were declining steadily in highly industrialized Western nations but remained persistently high in developing and newly industrialized countries [1,2].

Pathogenically, *H. pylori* is the first bacterium definitively linked to human malignancies, including gastric adenocarcinoma and gastric mucosa-associated lymphoid tissue lymphoma. Accumulating evidence indicates that *CagA*-positive strains of *H. pylori* play a crucial role in the neoplastic transformation of mammalian cells [2].

High-sensitivity detection of *H. pylori* has long been a focus of scientific research. Diagnostic methods can be classified as invasive or non-invasive. Invasive approaches include endoscopic evaluation, rapid urease testing, histologic examination, and bacterial culture. Non-invasive methods comprise the urea breath test (currently one of the most widely used non-invasive techniques), stool antigen tests, serology, and molecular diagnostics [3,4].

Among molecular diagnostic techniques, quantitative real-time PCR (qPCR) is commonly employed for *H. pylori* detection and represents one of the most frequently used methods for quantifying nucleic acids in biological and environmental samples [5]. This approach offers simple operation, relatively low cost, high sensitivity, and strong target specificity, making it suitable for large-scale screening in epidemiological studies [6].

However, conventional detection methods and qPCR both exhibit significant limitations. Traditional techniques often exhibit limited sensitivity and specificity, complex procedures, long turnaround times, and, in some cases, reliance on expensive instrumentation. Although qPCR is a mainstream molecular diagnostic tool, it requires sophisticated thermal cycling equipment and is time-consuming, rendering it particularly unsuitable for clinical testing in primary care or resource-limited settings.

To address the need for point-of-care diagnostics, highly sensitive isothermal amplification methods, which do not require costly instruments and are well-suited to field testing, have emerged as promising alternatives to classic PCR-based techniques. Among these, loop-mediated isothermal amplification (LAMP) has gained prominence in recent years due to its notable advantages [7,8]. This technique employs four to six specific primers and Bst polymerase to efficiently amplify target DNA at a constant temperature of 60–65 °C within 30–60 min [9,10]. LAMP reactions are rapid, highly specific, and do not depend on expensive instrumentation. The Bst polymerase most commonly used in LAMP exhibits strong strand displacement activity, eliminating the need for a separate DNA denaturation step. Moreover, because amplification occurs at a stable temperature, detection can be achieved much faster than with standard techniques, and in some cases, results can even be interpreted visually [11]. LAMP has been widely adopted for pathogen detection and field applications in resource-constrained environments.

However, the standalone use of isothermal amplification techniques carries significant risks of false-positive amplification due to cross-contamination, nonspecific amplification, or primer-dimer formation [12]. Concurrently, the CRISPR-Cas system, derived from the adaptive immune mechanism in bacteria, enables high-sensitivity detection of target sequences by cleaving fluorescently labeled probes to release signals [13,14,15]. It has now evolved into a cutting-edge technology in genome editing. Among the various CRISPR-Cas systems, Cas12b demonstrates considerable potential in molecular diagnostics owing to its strict target recognition requiring a specific protospacer adjacent motif (PAM), as well as its unique collateral cleavage activity, whereby the target-activated Cas12b non-specifically degrades nearby single-stranded DNA reporters to generate a detectable signal [16,17,18,19]. Currently, the integration of isothermal amplification with CRISPR/Cas systems, combining LAMP with CRISPR/Cas12b in particular, has become a prominent research focus due to its marked advantages in nucleic acid testing. This synergistic approach harnesses both efficient amplification and high-specificity recognition, thereby substantially improving the accuracy and applicability of *H. pylori* detection.

**Figure 1:**
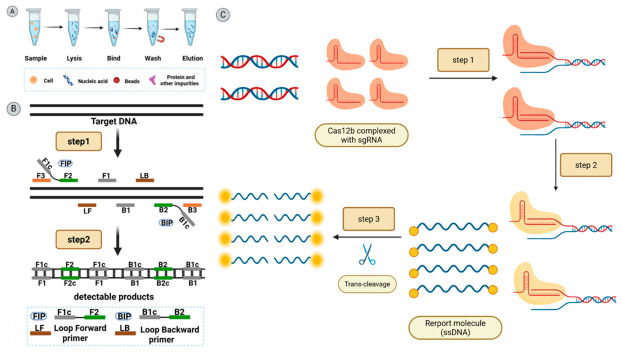
Schematic representation of CRISPR/Cas12b and LAMP detection. (**A**) Schematic diagram of nucleic acid extraction workflow, (**B**) schematic diagram of LAMP mechanism, and (**C**) schematic diagram of CRISPR/Cas12b detection mechanism.

Based on this background, the present study aims to establish a rapid *H. pylori* detection method based on LAMP-CRISPR/Cas12b, as illustrated in Figure 1. The method targets the clinically significant *CagA* gene, and through systematic optimization of LAMP primers, sgRNA, and reaction components, it builds a field-detection system compatible with portable devices or test strips. The anticipated results are expected to provide a novel technical pathway for rapid, non-invasive, and low-cost diagnosis of *H. pylori*, offering a practical solution for primary care settings and point-of-needs testing. This study presents the following innovative aspects:

① High Sensitivity and Specificity: The high specificity of the CRISPR/Cas12b system, combined with the efficient amplification capability of LAMP technology, significantly enhances detection accuracy, overcoming the limitations of individual techniques and demonstrating superiority over traditional PCR and culture methods.
② Fast and simple: The assay can be completed within one hour, significantly reducing the time required for conventional testing. It can be paired with a portable instrument, enabling users with minimal training to perform the test. This is particularly suitable for resource-limited settings, offering low equipment requirements and rapid diagnosis for point-of-care testing.
③ Non-invasive sampling: Patient stool samples can be utilized, eliminating the need for invasive specimen collection. This enhances patient comfort and compliance, making it particularly suitable for pediatric and elderly populations.

## 2. Materials and Methods

### 2.1. Reagents and Materials

The primary reagents utilized in this study were as follows: the Mag-MK Bacterial Genome DNA Extraction Kit (B518725-0050) was sourced from Sangon Biotech (Shanghai, China). Bst II Pro DNA Polymerase Large Fragment (P703-01) was obtained from Novozymes Biotechnology (Nanjing, China). dNTPs and LAMP fluorescent dye were purchased from New England Biolabs. Goldview nucleic acid dye (10,000×) was acquired from Yisheng Biotechnology (Shanghai, China). AapCas12b Nuclease (32118-01) was supplied by Tulugang Biotechnology (Shanghai, China). Agarose, 6× DNA loading buffer, DNA Marker (100–600 bp), de-ionized water (ddH_2_O), 50× TAE electrophoresis buffer, and primers were all procured from Sangon Biotech (Shanghai, China)**.** The *H. pylori* probe-based real-time fluorescence PCR kit was purchased from Geju Medical Technology (Hangzhou , China). The bacterial strains employed in this research were provided by Zhuzhou Central Hospital (Zhuzhou, China).

The key instruments applied in this investigation included a −80 °C ultra-low temperature freezer (Aucma, Qingdao, China), a high-speed microcentrifuge (Michael Laboratory Instrument, Changsha, China), a microspectrophotometer (Xinling Biotechnology, Shanghai, China), micropipettes (Eppendorf, Hamburg, Germany), a biosafety cabinet (Xinbeixi Biotechnology, Jinan, China), Real-time fluorescence quantitative PCR(qPCR) instrument (Bioer Technology, Hangzhou, China), an electronic balance (Puchun Instrument, Shanghai, China), an electrophoresis apparatus (Yixin Analytical Instrument, Shanghai, China), and a gel imaging system (Yixin Analytical Instrument, Shanghai, China).

### 2.2. Strain Cultivation and DNA Extraction

The recombinant plasmid pUC57-HP_CA, containing the target gene, was synthesized by Sangon Biotech (Shanghai, China). This quantified plasmid was subsequently used as the standard template for the LAMP-CRISPR/Cas12b detection system.

The *H pylori*, *Pseudomonas aeruginosa*, *Mycobacterium tuberculosis*, *Escherichia coli*, and *Staphylococcus aureus* strains used in this study were laboratory-preserved strains provided by Zhuzhou Central Hospital (Zhuzhou, China). To ensure biosafety, all bacterial strains were inactivated prior to nucleic acid extraction. Genomic DNA was extracted using the Mag-MK Bacterial Genome DNA Extraction Kit (B518725-0050, Sangon Biotech, Shanghai, China) according to the manufacturer’s instructions. The concentration and purity of the extracted DNA were measured using a microspectrophotometer (Xinling Biotechnology, Shanghai, China). Subsequently, the DNA samples were stored at −20 °C for subsequent specificity evaluation and performance analysis.

### 2.3. Design and Screening of LAMP Primers

The conserved sequence of the *H. pylori CagA* gene (Gene ID: AB015416.1) was obtained from the NCBI database. LAMP primers were designed using the online Primer Explorer V5 software ((https://primerexplorer.eiken.co.jp/e/ (accessed on 4 July 2025))). The designed primers were subjected to specificity analysis through the NCBI BLAST tool (version 6.02). The primer set showing the highest specificity was selected and synthesized by Sangon Biotech. The synthesized primers were then used in LAMP reactions. Amplification products were analyzed by 2% agarose gel electrophoresis to identify the primer set with the best amplification efficiency. The LAMP reaction was carried out in a 25 µL total volume, containing: 2.5 µL of 10 × Reaction Buffer 6, 1.5 µL of 100 mM Mg^2+^, 3.5 µL of 10 mM dNTPs, 4 µL of FIP/BIP primers (10 µM each), 0.5 µL of F3/B3 primers (10 µM each), 1 µL of Loop F primer (10 µM), 1 µL of Bst polymerase, 0.5 µL of LAMP fluorescent dye, 2 µL of template DNA, and nuclease-free water to bring the total volume to 25 µL. Reactions with nuclease-free water served as the negative control. Fluorescence was monitored in qPCR instrument at 64 °C, with readings taken every minute for 60 min.

### 2.4. Optimization of the LAMP Detection System

To optimize the detection system for *H. pylori* using LAMP, we systematically optimized key reaction components. Parameters varied included: reaction temperature (61 °C, 62 °C, 63 °C, 64 °C, 65 °C), Bst polymerase concentration (160 U/mL, 240 U/mL, 320 U/mL, 400 U/mL, 480 U/mL), betaine concentration (0 mM, 0.2 mM, 0.4 mM, 0.6 mM, 0.8 mM), dNTP concentration (1.2 mM, 1.4 mM, 1.6 mM, 1.8 mM, 2.0 mM), Mg^2+^ concentration (2 mM, 4 mM, 6 mM, 8 mM, 10 mM), and the ratio of inner to outer primers (1:2, 1:4, 1:6, 1:8, 1:10). A constant volume of 2 µL of DNA template was used for all reactions, and each optimization experiment included a negative control set. Real-time fluorescence monitoring was employed to assess amplification efficiency by analyzing amplification curve characteristics, time to positivity, and endpoint fluorescence intensity to define the optimal reaction conditions.

### 2.5. Sensitivity and Specificity Analysis of the LAMP Detection Method

Sensitivity Analysis: To evaluate the sensitivity of the LAMP-based assay for detecting the *H. pylori CagA* gene, a serial dilution of the standard template was prepared. The template was diluted in a geometric progression to concentrations ranging from 1 to 10 copies/µL. These dilutions served as the test templates and were assayed using the optimized reaction system. A blank control consisting of ddH_2_O was included. Real-time fluorescence curves were recorded. The sensitivity of the assay was determined by analyzing the characteristic shape of the amplification curve, the time to amplification, and the fluorescence intensity.

Specificity Analysis: To assess the specificity of the LAMP-based assay for the *H. pylori CagA* gene, a panel of four *CagA* gene-negative controls was established. Nucleic acids extracted from *Escherichia coli*, *Staphylococcus aureus*, *Mycobacterium tuberculosis*, and *Pseudomonas aeruginosa* were used as templates and assayed under the optimized conditions. A ddH_2_O blank control was included. Real-time fluorescence curves were recorded. The specificity of the assay was determined by analyzing the characteristic shape of the amplification curve, the time to amplification, and the fluorescence intensity.

### 2.6. Screening of sgRNAs

Based on the DNA sequence of the optimal LAMP primer amplification product, the Cas Guider v4.2 software developed by Wuhan Tech Biotech Co., Ltd. (Wuhan, China) was used for sgRNA design. The designed sgRNAs were subjected to specificity analysis using the BLAST tool on the NCBI website, and those with higher specificity were selected and synthesized by Guangzhou Aiji Biotechnology Co., Ltd. (Guangzhou, China). The synthesized sgRNAs were then employed in CRISPR/Cas12b reactions to identify the set with the highest amplification efficiency. The CRISPR/Cas12b reaction system, with a total volume of 20 µL, comprised 2 µL of 10 × HOLMES Buffer 1, 0.2 µM fluorescent probe, 0.25 µM sgRNA, 0.2 µM AapCas12b Nuclease, and 1 µL of template, with ddH_2_O added to reach the final volume. Using the LAMP product as the reaction template, 1 µL of the LAMP product was transferred into the CRISPR/Cas12b reaction system. Fluorescence values were measured using qPCR instrument at 55 °C every minute for 60 min.

### 2.7. Establishment and Optimization of the Two-Step LAMP-CRISPR/Cas12b Detection Method

In the two-step LAMP-CRISPR/Cas12b detection, the LAMP reaction is first carried out for pre-amplification to obtain the reaction substrate for AapCas12b. Then, its cleavage activity is used to generate fluorescence. Using DNA as the template for the LAMP reaction, after reacting at 64 °C for 30 min, 1 µL of the LAMP product is transferred to the CRISPR-Cas12b reaction system. qPCR instrument is used to read the fluorescence value every minute at 55 °C.

To obtain the optimal two-step LAMP-CRISPR/Cas12b reaction system for detecting *H. pylori*, we optimized the CRISPR/Cas12b reaction temperature (58 °C, 60 °C, 62 °C, 64 °C) and the LAMP time (6 min, 8 min, 10 min, 12 min, 14 min, 16 min) in sequence. The DNA template used in all experiments was 2 µL, and a set of negative controls was used for all optimizations. The real-time fluorescence values were detected to evaluate the fluorescence intensity under different reaction conditions. The shape of the amplification curve, amplification time, and fluorescence intensity were observed to determine the optimal reaction conditions.

### 2.8. Establishment and Optimization of the One-Step LAMP-CRISPR/Cas12b Detection Method

The one-step detection system has a total volume of 20 µL, and its components include 5 µL of 2 × LAMP master mix, 1 µL of 10 × HOLMES Buffer 1, 2 µL of 10 × LAMP primer mixture, 0.5 µM gRNA, 0.25 µM AapCas12b Nuclease, 1 µM ssDNA reporter, 1 µL of template, and ddH_2_O to make up to 20 µL. Among them, the components of the 2 × LAMP master mix include 10 µL of 10 × Reaction Buffer 6, 6 µL of 100 mM Mg^2+^, 14 µL of 10 mM dNTPs, 4 µL of Bst polymerase, and ddH_2_O to make up to 50 µL. A real-time fluorescence PCR instrument is used to read the fluorescence value every minute at 60 °C for 60 min.

To obtain the optimal one-step LAMP-CRISPR/Cas12b reaction system for detecting *H. pylori*, we optimized the ratio of 2 × LAMP master mix to 10 × HOLMES Buffer 1 (1×:1×, 1×:0.5×, 1×:0, 0.5×:1×, 0.5×:0.5×, 0.5×:0), the one-step reaction temperature (56 °C, 57 °C, 58 °C, 59 °C, 60 °C), the AapCas12b concentration (0.05 µM, 0.1 µM, 0.15 µM, 0.2 µM, 0.25 µM), and the ssDNA reporter (0.5 µM, 0.75 µM, 1 µM, 1.25 µM, 1.5 µM) in sequence. The DNA template used in all experiments was 1 µL, and a set of negative controls was set for all optimizations. A real-time fluorescence PCR instrument was used to read the fluorescence value every minute for 60 min. The fluorescence intensity under different reaction conditions was evaluated, and the shape of the amplification curve, amplification time, and fluorescence intensity were observed to determine the optimal reaction conditions.

### 2.9. Sensitivity Analysis of LAMP-CRISPR/Cas12b

To evaluate the sensitivity of the LAMP-CRISPR/Cas12b technology for detecting the *H. pylori CagA* gene, a series of concentration gradients was set according to the standard template concentration. The template was diluted proportionally to 1–10 copies/µL, and these were used as the detection templates. The optimized system was used for detection. The blank control group was ddH_2_O. The real-time fluorescence values were recorded. The shape of the amplification curve, amplification time, and fluorescence intensity were observed to determine the sensitivity of this detection technology.

### 2.10. Specificity Analysis of LAMP-CRISPR/Cas12b

To evaluate the specificity of the LAMP-CRISPR/Cas12b technology for detecting the *H. pylori CagA* gene, four groups of *H. pylori CagA* gene-negative groups were set. The nucleic acids of *Escherichia coli*, *Staphylococcus aureus*, *Mycobacterium tuberculosis*, and *Pseudomonas aeruginosa* were extracted as templates, and the optimized system was used for detection. The blank control group was ddH_2_O. The real-time fluorescence values were recorded. The shape of the amplification curve, amplification time, and fluorescence intensity were observed to determine the specificity of this detection technology.

### 2.11. Detection and Application of Clinical Samples

To comprehensively evaluate the feasibility of LAMP-CRISPR/Cas12b in detecting clinical samples, the DNA of 34 clinical samples was extracted. The one-pot detection system was used. The shape of the amplification curve, amplification time, and fluorescence intensity were observed, and the test results were statistically analyzed.

## 3. Results

### 3.1. Establishment of a LAMP Method for the Detection of H. pylori

#### 3.1.1. LAMP Primer Screening

**Figure 2:**
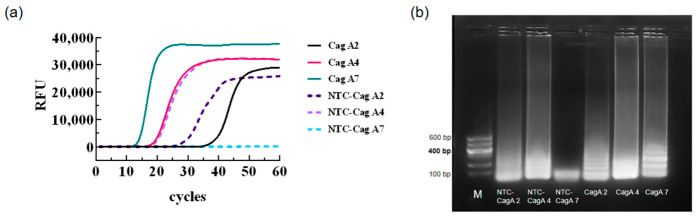
LAMP Primer Panel Screening. (**a**) Amplification curves for real-time LAMP; (**b**) agarose gel electrophoresis. CagA2/4/7: Positive controls for the 2nd, 4th, and 7th sets of *CagA* LAMP primers, respectively; NTC-CagA2/4/7: Negative controls for the 2nd, 4th, and 7th sets of *CagA* LAMP primers, respectively.

*H. pylori* genomic DNA was selected as the positive template, while ddH_2_O served as the blank template control for LAMP primer screening. As shown in Figure 2, when amplifying with *H. pylori* genomic DNA using primers from groups 1 to 7, ladder-like bands were observed in all cases. However, when ddH_2_O was used as the template, only the seventh primer group failed to produce ladder-like bands. The ladder-like bands amplified by the seventh primer set were distinct, with minimal primer dimer formation. Consequently, the seventh primer set was selected as the LAMP reaction primers, with their sequences presented in Table 1.

#### 3.1.2. Optimization of LAMP Reaction Conditions

**Figure 3:**
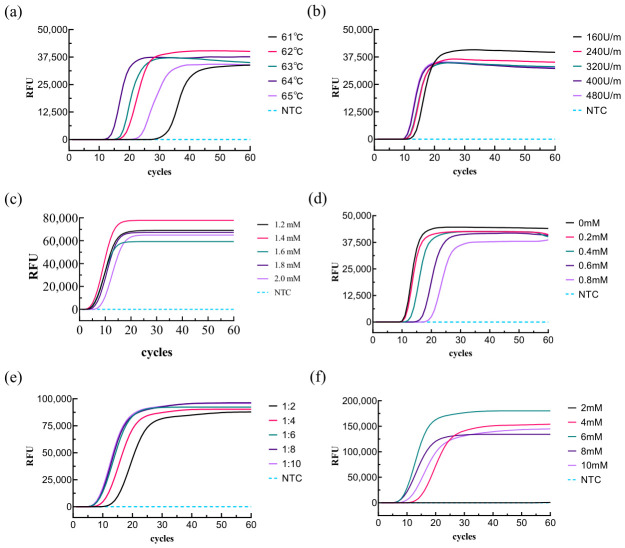
Optimization of the LAMP Reaction System. (**a**) Temperature optimization of the LAMP reaction; (**b**) Enzyme concentration optimization of the LAMP reaction; (**c**) dNTP concentration optimization of the LAMP reaction; (**d**) betaine concentration optimization of the LAMP reaction; (**e**) primer ratio optimization of the LAMP reaction; and (**f**) magnesium ion concentration optimization of the LAMP reaction.

Since the reaction system influences detection time, sensitivity, and specificity, we optimized key components of the LAMP reaction to establish an optimal reaction system. Results are shown in Figure 3. The highest LAMP efficiency was achieved at a reaction temperature of 64 °C (Figure 3a), a Bst polymerase concentration of 320 U/mL (Figure 3b), a dNTP concentration of 1.4 mM (Figure 3c), a betaine concentration of 0 mM (Figure 3d), an internal-to-external primer ratio of 1:8 (Figure 3e), and a Mg^2+^ concentration of 8 mM (Figure 3f). The optimized positive reaction exhibited a start time of 7 min, with the fluorescence curve reaching a plateau within 30 min. No non-specific amplification occurred under these optimized conditions. In summary, we established a stable and highly efficient LAMP reaction system.

### 3.2. Establishment of a LAMP-CRISPR/Cas12b Method for Detection of H. pylori

#### 3.2.1. sgRNA Screening

**Figure 4:**
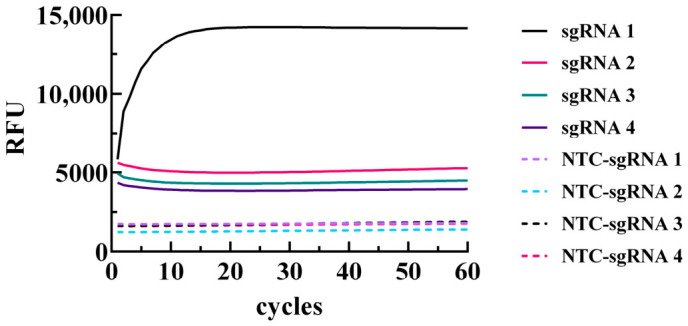
sgRNA Screening.sgRNA1/2/3/4: Positive controls for the 1nd, 2th, 3th,4th sets of sgRNA, respectively; NTC-sgRNA1/2/3/4: Negative controls for the 1nd, 2th, 3th,4th sets of sgRNA, respectively.

Four sgRNAs were designed based on the sequence of the LAMP-amplified DNA fragment. The corresponding DNA fragment obtained via LAMP served as the positive template, while ddH_2_O acted as the blank template control for sgRNA screening. Reactions were performed using each of the four designed sgRNAs to identify the optimal one. As shown in Figure 4, fluorescence was observed in the reaction tube containing sgRNA-1, while no significant fluorescence was detected in the other tubes. Therefore, sgRNA-1 was selected for subsequent experiments in this study. The sequence of sgRNA-1 is provided in Table 2.

#### 3.2.2. Optimization of the LAMP-CRISPR/Cas12b Two-Step Detection Method

**Figure 5:**
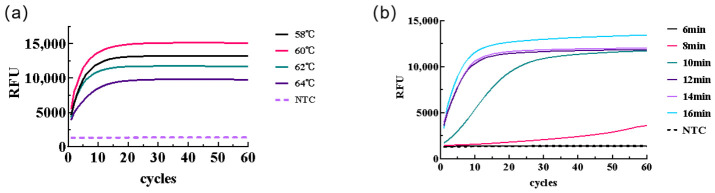
Optimization of the LAMP-CRISPR/Cas12b two-step detection method. (**a**) Reaction temperature optimization; (**b**) LAMP time optimization.

Optimization of the reaction temperature was conducted using the selected sgRNAs, with four temperature gradients established to determine the optimal reaction temperature. Results indicated (Figure 5a) that fluorescence curves were generated at all four temperatures, but fluorescence intensity peaked at 60 °C. Therefore, 60 °C was selected for subsequent experiments.

Based on the optimized conditions above, the LAMP reaction time was further optimized by testing six different reaction durations to determine the optimal time. The optimization reactions were performed at a constant temperature of 60 °C. Results showed (Figure 5b) that a 10 min reaction was insufficient to produce a distinct fluorescence curve, whereas a 12 min reaction yielded a clear curve. Consequently, the LAMP reaction time for subsequent experiments was set to 15 min to enhance efficiency while avoiding reduced sensitivity caused by shortening the amplification time.

#### 3.2.3. Optimization of the LAMP-CRISPR/Cas12b One-Step Detection Method

**Figure 6:**
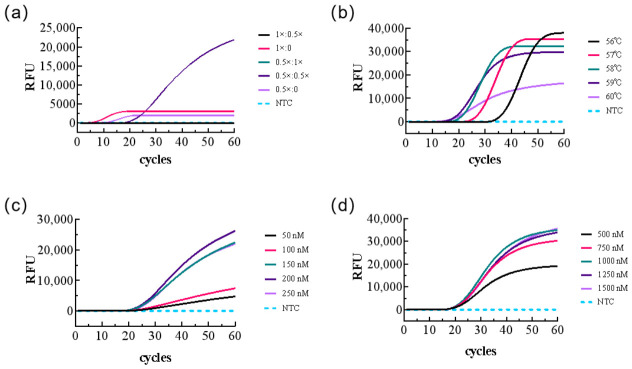
Optimization of the LAMP-CRISPR/Cas12b one-step detection method. (**a**) Optimization of the ratio between LAMP master mix and CRISPR/Cas12b reaction buffer; (**b**) optimization of reaction temperature; (**c**) optimization of AapCas12b protein concentration; and (**d**) optimization of ssDNA reporter concentration.

Due to the operational complexity and susceptibility to aerosol contamination associated with two-step LAMP-CRISPR/Cas12b, we aimed to establish a one-tube detection system. As shown in Figure 6a, combining 0.5 × LAMP master mix with 0.5 × CRISPR buffer (10 × HOLMES Buffer 1) yielded optimal detection performance. Reaction temperatures were tested between 56 °C and 60 °C. Results indicated that while 60 °C yielded the fastest positive results, it produced lower detection fluorescence values. Considering the requirement for rapid and efficient detection, 59 °C was selected as the optimal reaction temperature (Figure 6b). For Cas12b and ssDNA reporter concentrations, the highest fluorescence values were observed at 0.2 µM and 1 µM, respectively, with no nonspecific fluorescence detected in negative controls (Figure 6c,d). In summary, our established one-step LAMP-CRISPR/Cas12b *H. pylori* diagnostic assay operates at 59 °C, with a 20 µL reaction volume comprising: 5 µL of 2 × LAMP master mix, 1 µL of 10 × HOLMES Buffer 1, 2 µL of 10 × LAMP master mix, 0.25 µM sgRNA, 0.2 µM AapCas12b, 1 µM ssDNA reporter, 1 µL of template, and ddH_2_O to a final volume of 20 µL.

### 3.3. Sensitivity Analysis of LAMP-CRISPR/Cas12b Detection

**Figure 7:**
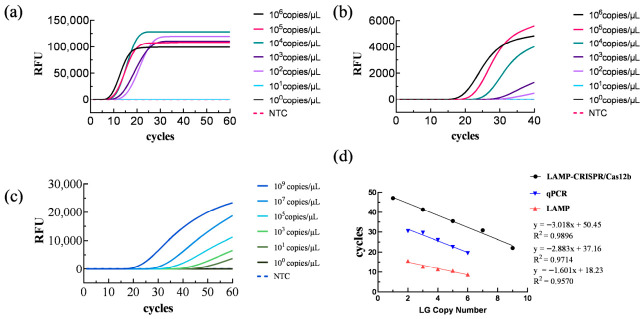
Sensitivity Analysis. (**a**) LAMP detection sensitivity analysis; (**b**) qPCR detection sensitivity analysis; (**c**) LAMP-CRISPR/Cas12b one-step detection sensitivity analysis; and (**d**) correlation analysis of the three detection methods.

Using *H. pylori* genomic DNA diluted in 10-fold serial dilutions as template, LAMP, PCR, and one-step LAMP-CRISPR/Cas12b assays were performed. The results are shown in Figure 7. When genomic DNA extracted from *H. pylori* cultures at concentrations ranging from 3.14 × 10^6^ to 3.14 × 10^2^ copies/µL was used as the detection template, both LAMP and PCR reactions exhibited elevated fluorescence curves. However, no significant fluorescence was observed in the reaction systems when genomic DNA from *H. pylori* at a concentration of 3.14 × 10^1^ copies/µL or ddH_2_O was used as template. The one-step LAMP-CRISPR/Cas12b assay achieved a minimum detectable concentration of 3.14 × 10^1^ copies/µL, indicating superior sensitivity of this system.

### 3.4. Specificity Analysis of LAMP-CRISPR/Cas12b Detection

**Figure 8:**
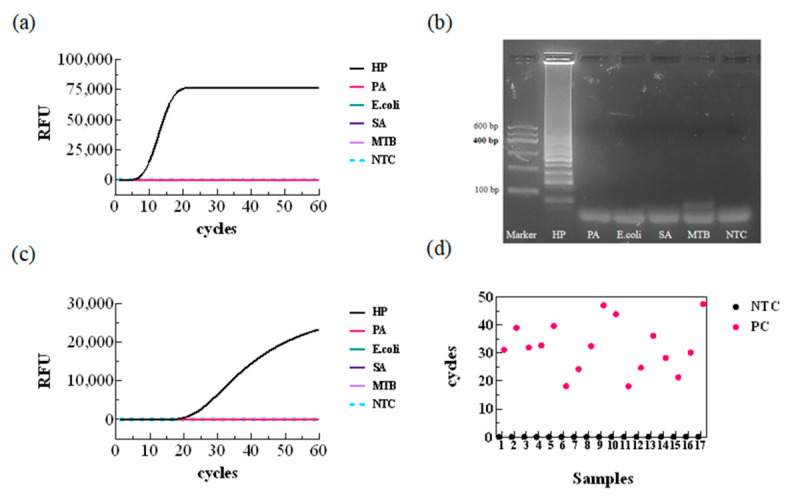
Specificity Analysis and Clinical Sample Detection. (**a**) LAMP detection specificity analysis; (**b**) agarose gel electrophoresis; (**c**) LAMP-CRISPR/Cas12b one-step detection specificity analysis; and (**d**) clinical sample detection. PA—*Pseudomonas aeruginosa*; E.coli—*Escherichia coli*; SA-*Staphylococcus aureus*; MTB-*Mycobacterium tuberculosis*; PC—Positive Control; NTC—No Template Control.

Using an optimized LAMP system, five different pathogens, including *H. pylori*, were used as template DNA for LAMP and LAMP-CRISPR/Cas12b one-step specific detection. As shown in Figure 8, both the LAMP assay and the one-step reaction yielded distinct fluorescence curves and amplification bands only for *H. pylori*, demonstrating the system’s high specificity against the tested non-target pathogens.

### 3.5. Clinical Sample Detection and Applications

The LAMP-CRISPR/Cas12b assay was performed on seventeen culture-positive samples and seventeen culture-negative samples in parallel, and the fluorescence amplification onset time was plotted as a heatmap. The results (Figure 8d) showed that none of the negative samples exhibited significant changes in fluorescence intensity, while all positive samples triggered amplification, achieving 100% concordance with the reference methods. The sensitivity and specificity of the LAMP-CRISPR/Cas12b assay for clinical samples were both 100%, with no false-positive or false-negative results observed, indicating the reliability of this reaction system for *H. pylori* detection.

## 4. Discussion

It is discussed that *H. pylori* has a wide range of infection and great harm, and it is an important pathogenic bacterium for malignant tumors such as gastric cancer. Driven by the demand for rapid and accurate diagnosis, the detection of *H. pylori* has transitioned from traditional culture-based approaches through conventional PCR and isothermal amplification (RPA/LAMP), advancing to the cutting-edge CRISPR/Cas technology. Jalalypour et al. [20] used invasive detection methods to perform PCR and tissue culture methods on gastric mucosa specimens. The PCR method has higher specificity (94.44%) and sensitivity (88.24%) than the tissue culture method. However, although PCR has strong specificity, it requires a complex thermal cycling temperature-changing process and subsequent gel electrophoresis analysis, which limits its application in resource-limited settings. The *H. pylori* LAMP detection platform established by Bakhtiari et al. [21] has high sensitivity (100%) and specificity, and can be used as a sensitive, specific, and economical method for the identification of *H. pylori* in clinical samples. However, there are problems such as false positives when using LAMP alone [22]. The CRISPR/Cas12a detection platform established by Broughton et al. [23] can complete the detection of SARS-CoV-2 from the RNA extract of respiratory swabs in 40 min. Although the sensitivity and specificity are better than the traditional methods, it is difficult to be compatible with sensitivity, specificity, and portability at the same time, and it is difficult to adapt to on-site rapid detection [24,25].

In this study, a one-pot LAMP-CRISPR/Cas12b detection system for *H. pylori* was successfully constructed, which has significant advantages. First of all, the LAMP technology has the characteristic of isothermal amplification. The reaction can be completed only at a constant temperature, getting rid of the dependence on a precise thermal cycler, and greatly reducing the equipment cost and operation difficulty [26]. Secondly, by introducing the CRISPR/Cas12b detection technology, the false-positive problem easily caused by aerosol pollution in the single LAMP reaction is effectively avoided, and the pollution risk caused by the open-lid operation in the two-step LAMP-CRISPR detection is also solved [27,28,29]. However, when exploring the one-pot detection system, there is a common situation where the LAMP reaction system and the CRISPR/Cas12b reaction system are incompatible. Because the standard LAMP buffer and CRISPR/Cas12b buffer have distinct compositions and different optimal ionic environments, simply combining the two systems in one tube would lead to component interference and an imbalanced reaction environment, ultimately causing assay failure. To overcome this incompatibility, we carefully adjusted the mixing ratio of the LAMP master mix and the CRISPR reaction buffer to balance the reaction conditions for both the amplification and cleavage steps, thereby successfully establishing the one-pot system.

Temperature is also an important factor affecting the reaction efficiency. The LAMP reaction temperature is higher than the CRISPR/Cas12b reaction temperature. Therefore, we optimized the one-step reaction temperature. Since LAMP plays a leading role in the early stage of the reaction, as the temperature rises, the starting time of the positive reaction shortens. However, the final fluorescence value of the reaction gradually decreases. It is worth noting that when the reaction temperature reaches the optimum temperature of CRISPR/Cas12b, the fluorescence signal value is at a low level. This observation was unexpected, as we initially hypothesised that the highest fluorescence signal would occur at the Cas12b-optimal temperature. We speculate that this may be because the change in temperature affects the enzymatic cleavage activity of Cas12b or the binding efficiency of sgRNA rather than simply due to too fast cleavage. This explanation is plausible and consistent with the known temperature sensitivity of Cas enzymes. In summary, we selected 59 °C as the subsequent reaction temperature.

In addition, other components in the reaction system were also optimized in this study to ensure high sensitivity and specificity, enabling robust detection of *H. pylori* at low concentrations (as low as 3.14 × 10^1^ copies/µL, compared with 3.14 × 10^2^ copies/µL for stand-alone LAMP and PCR). These optimisation results are logical and follow the expected trends. This one-step detection mode is simple, fast, and requires minimal operator expertise. It is well-suited for promotion in primary health care institutions or resource-poor areas, providing an efficient and reliable point-of-care testing tool for early screening and epidemiological investigation of *H. pylori* infection [30].

Despite these promising findings, several limitations should be acknowledged. First, large-scale clinical sample testing has not yet been performed; the current evaluation was based on a synthetic plasmid and a limited set of bacterial strains, and therefore, the assay’s performance in authentic clinical specimens (e.g., gastric biopsies or stool samples) remains to be validated. Second, the specificity panel included only four additional bacterial species; a broader range of gastrointestinal pathogens and commensals would further confirm specificity. Third, the long-term storage stability of the one-pot reaction mix has not been assessed, which is important for practical deployment. Regarding the “rapid” nature of the test, although the current turnaround time is as short as 1 h, further optimisation could focus on reducing the reaction time to 30–40 min by fine-tuning the polymerase concentration, primer design, or adopting ultra-fast thermal management. Additionally, integrating the one-pot assay into a microfluidic or paper-based device could simplify the workflow and reduce hands-on time, making it even more suitable for true point-of-care settings in resource-limited environments. Future research should therefore prioritise large-scale clinical validation, expand specificity testing, evaluate lyophilisation or room-temperature stabilisation, and explore strategies to further shorten the detection time while maintaining sensitivity and specificity.

## 5. Conclusions

This study combines the dual advantages of CRISPR/Cas12b and LAMP technologies to achieve efficient amplification and high-specificity recognition, significantly improving the accuracy and application scope of *H. pylori* detection. The method is accurate and sensitive, with a detection limit of 3.14 × 10^1^ copies/µL, excellent specificity, and simple operation. The entire assay can be completed within 60 min. At the same time, the one-pot detection method can effectively avoid the risk of aerosol pollution. In addition, equipped with a portable detection device, it can provide an efficient and feasible technical solution for the rapid screening of pathogens in grass-roots medical institutions and resource-poor areas, and has good clinical transformation potential and application prospects [31,32].

## Figures and Tables

**Table 1:** LAMP primer sequences used in this study.

Primer Set	Primer	Sequence (5′→3′)
Primer Set 1	F3-1	CATACAACCCCCTATCCTT
	B3-1	CCAATCCCCACCAGTAG
	FIP-1	CCGTTCGGATTTGATTCCCTATGATGATAAAGAGAAAGCGGA
	BIP-1	GTTCATGGGCGTGTTTGATGGCTCTCCATTTTTTTCTGC
	LF-1	CCTGCAAAAGATTGTTTGGCAGA
Primer Set 2	F3-2	ACAACCCCCTATCCTTG
	B3-2	CCAATCCCCACCAGTAG
	FIP-2	CCGTTCGGATTTGATTCCCTATATGATAAAGAGAAAGCGGAG
	BIP-2	GTTCATGGGCGTGTTTGATGGCTCTCCATTTTTTTCTGC
	LF-2	CCTGCAAAAGATTGTTTGGCAGA
Primer Set 3	F3-3	ACAACCCCCTATCCTTG
	B3-3	CCAATCCCCACCAGTAG
	FIP-3	CCGTTCGGATTTGATTCCCTATTGATAAAGAGAAAGCGGAGT
	BIP-3	GTTCATGGGCGTGTTTGATGGCTCTCCATTTTTTTCTGC
	LF-3	CCTGCAAAAGATTGTTTGGCAGA
Primer Set 4	F3-4	ACAACCCCCTATCCTTG
	B3-4	CCAATCCCCACCAGTAG
	FIP-4	CCGTTCGGATTTGATTCCCTATGATAAAGAGAAAGCGGAGTT
	BIP-4	GTTCATGGGCGTGTTTGATGGCTCTCCATTTTTTTCTGC
	LF-4	CCTGCAAAAGATTGTTTGGCAGA
Primer Set 5	F3-5	TCTCTAAGGAAACGAGAGC
	B3-5	CAATTCCCTTTTGATGCCTT
	FIP-5	TGTTAGCTTGAGCTTTTGCTTCCAAGAAGTAGAGAAAAAATTGGAGAG
	BIP-5	GCCAAAAAGATGAGATTTTTGCGTCTGAGCGTAAGCGATTGC
	LB-5	ATAAAGAGGCTAATAGAGACGCAAG
Primer Set 6	F3-6	TCTCTAAGGAAACGAGAGC
	B3-6	GTCTTTCAAATTCTTGTTGACAT
	FIP-6	CTGTTAGCTTGAGCTTTTGCTTCAGAAGTAGAGAAAAAATTGGAGA
	BIP-6	AAGAGGCTAATAGAGACGCAAGAACAATTCCCTTTTGATGCC
	LB-6	GCAATCGCTTACGCTCAGAA
Primer Set 7	F3-7	AGAGAAAAAATTGGAGAGCAA
	B3-7	GTCTTTCAAATTCTTGTTGACAT
	FIP-7	CGCAAAAATCTCATCTTTTTGGCTCGGCAACAAAAATAAAATGGAAG
	BIP-7	AAGAGGCTAATAGAGACGCAAGAAGACAATTCCCTTTTGATGC
	LB-7	GCAATCGCTTACGCTCAGAA

**Table 2:** sgRNA and ssDNA reporter sequences used in this study.

Group	Sequence Name	Sequence (5′→3′)
sgRNA	sgRNA-1	TAATACGACTCACTATAGGGGTCTAGAGGACAGAATTTTTCAACGGGTGTGCCAATGGCCACTTTCCAGGTGGCAAAGCCCGTTGAGCTTCTCAAATCTGAGAAGTGGCACTCAGACAATTCCCTTTTGATGCC
	sgRNA-2	TAATACGACTCACTATAGGGGTCTAGAGGACAGAATTTTTCAACGGGTGTGCCAATGGCCACTTTCCAGGTGGCAAAGCCCGTTGAGCTTCTCAAATCTGAGAAGTGGCACGAGAGCAAAAGCGGCAACAAAAA
	sgRNA-3	TAATACGACTCACTATAGGGGTCTAGAGGACAGAATTTTTCAACGGGTGTGCCAATGGCCACTTTCCAGGTGGCAAAGCCCGTTGAGCTTCTCAAATCTGAGAAGTGGCACAAGTTTATCAGACAATTCCCTTT
	sgRNA-4	TAATACGACTCACTATAGGGGTCTAGAGGACAGAATTTTTCAACGGGTGTGCCAATGGCCACTTTCCAGGTGGCAAAGCCCGTTGAGCTTCTCAAATCTGAGAAGTGGCACAAATTCTTGTTGACATTTTCAA
Probe		FAM-TTTTTTTTTTTT-BHQ1

## Data Availability

The original contributions presented in this study are included in the article. Further inquiries can be directed to the corresponding author.

## Abbreviations

The following abbreviations are used in this manuscript:

HP	*Helicobacter Pylori*
LAMP	Loop-Mediated Isothermal Amplification
CRISPR	Clustered Regularly Interspaced Short Palindromic Repeats
Cas12b	CRISPR-Associated Protein 12b
*CagA*	Cytotoxin-Associated Gene A
PCR	Polymerase Chain Reaction
MALT	Mucosa-Associated Lymphoid Tissue
qPCR	Quantitative Polymerase Chain Reaction
Bst	Bacillus Stearothermophilus
dNTPs	Deoxyribonucleotide Triphosphates
ddH_2_O	Double-Distilled Water
FIP	Forward Inner Primer
BIP	Backward Inner Primer
F3	Forward Outer Primer
B3	Backward Outer Primer
LF	Loop Forward Primer
sgRNA	Single Guide RNA
ssDNA	Single-Stranded DNA
RPA	Recombinase Polymerase Amplification
RUT	Rapid Urease Test
ELISA	Enzyme-Linked Immunosorbent Assay
PA	*Pseudomonas aeruginosa*
E.coli	*Escherichia coli*
SA	*Staphylococcus aureus*
MTB	*Mycobacterium tuberculosis*
NTC	No Template Control
PC	Postive Control
